# Sudden challenges in teaching ecology and aligned disciplines during a global pandemic: Reflections on the rapid move online and perspectives on moving forward

**DOI:** 10.1002/ece3.7090

**Published:** 2021-01-23

**Authors:** Karen L. Bacon, Julie Peacock

**Affiliations:** ^1^ Botany and Plant Sciences School of Natural Sciences National University of Ireland Galway Ireland; ^2^ School of Geography University of Leeds Leeds UK

**Keywords:** ecology, fieldwork, laboratory teaching, online learning, Teaching online, undergraduate students

## Abstract

The challenges facing higher education in response to COVID‐19 are significant and possibly none more so than in ecology and aligned disciplines. Not only did most ecology lecturers have to rush lectures and tutorials online, but also laboratory and field classes. We reflect on our experience of this move and also consider those of 30 other ecology‐aligned teaching academics to summarize the challenges faced in the move online early in 2020 and the developing plans for adapting ecology teaching and learning going into the 2020/21 academic year. The move online had the most significant impact on field classes, with more of these canceled than lectures or laboratory classes. Most respondents to an online poll also highlighted that many respondents (~45%) felt that ecology was more impacted by COVID‐19 that even other STEM disciplines. The availability of technological solutions is key to moving forward and will hopefully enhance the teaching and learning experience for many beyond the current crisis.

## Graphical abstract

The challenges facing higher education in response to COVID‐19 are significant and possibly none more so than in ecology and aligned disciplines. Not only did ecology lecturers have to rush lectures and tutorials online, but also laboratory and field classes. We present our experience of this move and also considerations of 30 other ecology‐aligned teaching academics to summarize the challenges faced in the move online early in 2020 and the developing plans for adapting ecology teaching and learning going into the 2020/21 academic year.

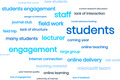

## 1 INTRODUCTION

Severe acute respiratory syndrome coronavirus 2 (SARS‐CoV‐2), more commonly referred to as COVID‐19 (WHO, 2020), brought a rapid change to teaching and learning in higher education around the world. With many programmes of study brought online and some canceled or significantly curtailed. The move to online teaching and learning represented a major challenge to higher education, particularly given the emergency nature of the move and the extremely restricted timeline available to university teaching staff to make major changes to their courses.

Online learning has been widely available to higher education learners since the 1990s, and distance learning was developed by Universities 100 years before that (Raven, 2003), and the pedagogic surrounding these forms has been rapidly developing and adapting to changes in technology (Stickney et al., 2019). Blended learning with a mixture of online and face‐to‐face teaching will be familiar to most teaching in higher education (Su, 2019). However, for many university teachers, and learners globally, the move to primarily or entirely online teaching and learning triggered directly due to the COVID‐19 pandemic in early 2020 was the first time that they fully engaged with online teaching and learning. This transition can have a very steep learning curve (Appana, 2008). Between January and April 2020, universities around the world began to restrict the physical presence of both staff and students with many ceasing in person activities almost entirely. In many cases, little notice was given that higher education institutions were closing as countries made the decision to enter various forms of “lockdown,” or restricted activity and travel. This move should not be thought of as traditional online teaching and learning, which takes time and careful planning and decision‐making, but as emergency remote teaching, reflecting a temporary shift in the teaching delivery (Hodges et al., 2020; Youmans, 2020).

The sudden pivot to online teaching represented a challenge and increase in workload for many staff in higher education. Although online learning has previously opened up opportunities to many students worldwide and increased accessibility (Appana, 2008), this particular shift exacerbated inequalities among students in many cases. Students who had not planned to study at home suddenly no longer had access to university spaces and may have faced challenges such as increased care responsibilities, work, or competition for technological resources (e.g., only one laptop shared between multiple users in the home) as well as unequal access to good Internet depending on their resources and home location, these issues have also been found by Burnett et al. (2020) with students studying in USA and Hallal et al. (2020) teaching students in Lebanon. For ecology and aligned disciplines, the move online had additional challenges. Most science, technology, engineering, and mathematics (STEM) degrees have significant components of laboratory classes, and many, including ecology, also have key fieldwork components. This work looks at the changes and challenges that occurred in ecology‐focused disciplines immediately after lockdown, and how plans may develop into the next academic year from the perspective of working in an Irish (National University of Ireland, Galway (NUIG), and a UK (University of Leeds (UoL)‐based university). We discuss the different approaches and the pedagogical issues for teaching ecology based on our own personal experiences. In addition, we consider informally supplied input from colleagues in other universities and countries that was gathered via Twitter and a GoogleForms document (hereafter referred to as the “online poll”), to which 30 people from the UK, Ireland, USA, South Africa, France, and Portugal responded.

As all academic disciplines have been impacted by COVID‐19 restrictions, is there a need to focus on the impact of ecological teaching specifically? Among the findings of our online poll was that nearly 45% (13 individuals) of respondents felt that ecology and aligned disciplines were impacted more than other STEM disciplines and only one responded felt that the subjects were less effected that others in STEM. This illustrates apprehension among ecology educators that the discipline has been and will be seriously impacted by the global pandemic with key concerns about laboratory and, particularly, field classes as both are integral to teaching and learning in ecology.

## 2 LECTURES

Lectures remain the primary means of delivering content to students in STEM (and may other) subjects, and most courses will have at least some lecturing component (Bligh, 2000; Nordlund, 2016). The changes and challenges discussed below will relate to more than just ecology and aligned disciplines but they also represent a very important component of teaching within these disciplines.

### 2.1 Lectures: Immediate changes

**FIGURE 1:**
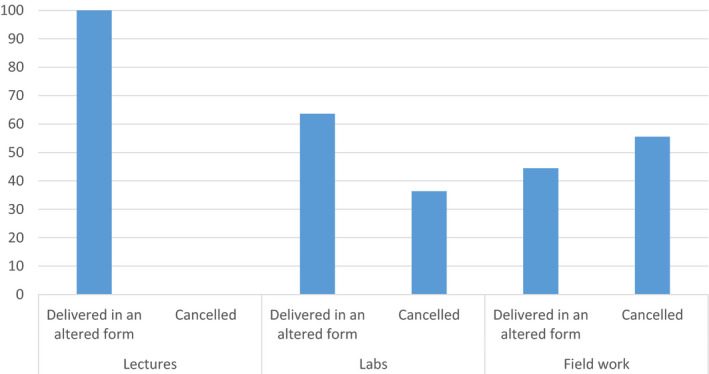
Impact of university shift to online teaching on lectures, labs and field classes from the online poll (total *n* = 26; when “not applicable” is removed number of responses is as follows: Lecture *n* = 23; Lab *n* = 11; Field work *n* = 18)

Of the 30 respondents in our online poll, none reported canceling lectures outright (Figure 1) during the move online, although ~45% of our respondents stated that they considered pedagogy during the move online. As highlighted by one of our reviewers, it is important to consider bias in our poll respondents. We consider one of the largest biases in our poll to be that our respective Twitter followings are more likely to include people with an interest in pedagogy who may have been more likely to respond to our call for information. This suggests that our finding of 45% of people considering pedagogy at this time is probably an overestimate. Several of these individuals noted that they always refer to pedagogy when preparing teaching and others referred to advice from senior colleagues or heads of teaching. Of the 55% who did not consider pedagogy, 55% stated that this was due to a lack of time, while two respondents (7%) stated that they had no decision‐making power in the move online and a further two stated that they were unsure of why this would be helpful.

Perhaps the simplest method of moving online was to post existing slides on the university virtual learning environment (VLE). For some staff, this may have been all that was possible due to time constraints, their own Internet access at home and insecurity of adding anything new to their existing system. For others, a big question was whether lectures should stick to the timetable and be delivered live, or become asynchronous. A brief straw poll of Ecology & Conservation students in NUIG (final year module with a class of 42) indicated a strong preference for asynchronous delivery (~71% of students) of voice‐over PowerPoints. This was due to a range of concerns including Internet accessibility and other responsibilities (e.g., caring for siblings or increased paid employment hours) and illustrating how using live sessions could unintentionally disadvantage some students. Among those who responded to our call for experiences in ecology teaching during the pandemic, all kept lectures running (except individuals who were not lecturing at the time). One respondent noted that they ran lectures online but that was as planned, so with no actual change. For the others, 62% reported using a mix of live and asynchronous sessions, 23% used fully asynchronous sessions, and 12% used entirely live session. Feedback on asynchronous lectures at NUIG was positive, and students highlighted the flexibility that it provided and appreciated being able replay lectures. Among lecturers who moved to online lecture delivery 50% referred to concerns or observations about loss of interaction between lecturer and students, lack of student feedback, and loss of questions within the lecture session. One respondent highlighted that online teaching is less fulfilling for some lecturers and stated “I'm concerned it will continue to be a lonely/isolating experience being a lecturer if we don't get to have actual contact with our students.”

In live sessions, 40% of respondents raised concerns about student engagement and eight respondents noted that students were apparently less engaged in online sessions. One respondent noted that students did not want to turn their cameras on in live sessions, “Students do not seem to like engaging online; they often do not want their cameras turns on. This makes it really hard to 'read the room' and teach effectively,” making it very difficult to engage with them even in a live session. Contrastingly, one respondent said that they felt more relaxed teaching online, “I'm much more relaxed working online and sharing my screen. I found it really helped me to slow down and take time to explain concepts,**”** highlighting that different methods of teaching work for different individuals. The majority of respondents, however, viewed online lecturing as a “fix” but far from ideal and felt that the negatives out‐weighted the positives of teaching in person. Whether lectures will become more confident and therefore more positively disposed to online lecturing, remains to be seen. Feedback from students at UoL was positive with students feeling connected to the University through these sessions and appreciated the clear enthusiasm from the lecturer.

#### 2.1.1 Changes for next year and beyond

The majority of those who responded to the online poll plan to make at least moderate changes to how they taught immediately after the lockdown. Approximately 55% said that they did not consider pedagogy in the initial move online, but 63% (10 individuals) of these respondents are planning to do so a moderate amount in future and only one respondent was negative to the idea of considering pedagogic literature to help inform teaching. Of those who did consider pedagogy during the move online, they were more likely to intend to make major changes (62%) to teaching and 63% also intended to consider pedagogy a lot in the coming academic year. This suggests that this lack of engagement with pedagogy (~55%) was motivated by the emergency nature of the response, rather than a lack of willingness to engage with educational theory. Lectures are likely to face at least moderate changes in many institutions next year, with many, including both NUIG and UoL requiring asynchronous online lectures. Of the 27 people who provided answers on how they would alter lectures in the next academic year, 30% stated an intention to split lectures into smaller chunks, for example, 15–25 min, to map onto student attention spans (Davis, 1993; Svinicki & McKeachie, 2013), although some more recent studies dispute this limit on students’ attention (Bradbury, 2016; Bunce et al., 2010). Asynchronous, recorded lectures are likely to have the additional benefit of providing a further support to dyslexic students and students with other specific learning requirements (Leadbeater et al., 2016). The second most common response was to focus on increasing interactions with students (26%) and to utilize tutorials (17%) to follow‐up students’ progress with asynchronous lectures. In general, it appears that many universities will move to asynchronous lectures, at least in part, with live tutorials as a supplement to this content. This represents a fairly significant change (and increase in workload) for many lecturing staff. Additionally, for lectures, use of tools such as Blackboard Ally, which provides automatically produced alternative formats of online material, will enhance accessibility for students. This will be incorporated into the Ecology & Conservation course in NUIG, for example, for next year.

The shift to asynchronous lectures should aid in enhancing accessibility, an essential consideration for the move online, as this makes students less dependent on having a strong and reliable internet connection (Figure 1). This was raised as a concern by seven respondents to our poll with one respondent noting that they had to abandon “a couple” of sessions due to connectivity problems and another highlighting that variable Internet “widens the socio‐economic divide” between students.

**FIGURE 2:**
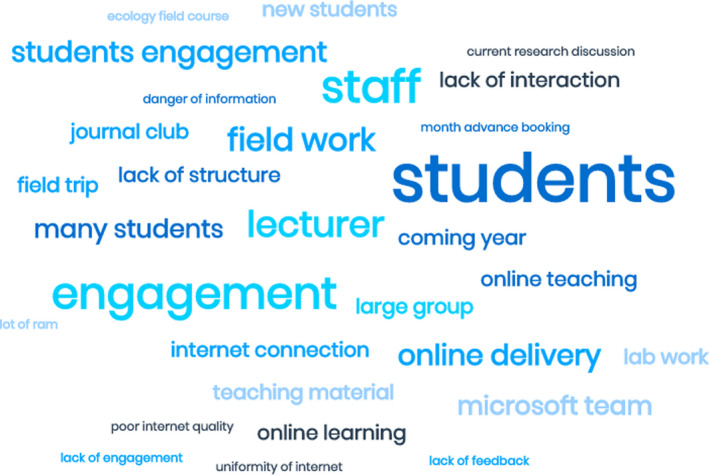
The 30 most commonly used words in all of the open text poll questions and clearly highlights that the focus is on students, staff/lecturers, engagement, and fieldwork

Figure 2: Suggested methods of interaction depending on urgency/immediacy and bandwidth of available internet. Image Credit. Bandwidth Immediacy Matrix. Daniel Stanford, 2020. Licensed under CC. Available from: https://www.iddblog.org/videoconferencing‐alternatives‐how‐low‐bandwidth‐teaching‐will‐save‐us‐all/

This will remain a potential problem for live sessions. Additionally, lecturers need to ensure that slides and documents can be easily read by screen readers (e.g., downloading the accessible pdf version for papers) and that fonts and backgrounds do not reduce legibility for visually impaired or dyslexic students. While this is standard practice in some institutions, in others this will be a new consideration for lecturers and support from institutions will be required. Students with disabilities will be greatly impacted by the lack of physical support usually provided by universities and lecturers need to try to ensure that, at least, the material available to students is not actively furthering this disadvantage. As one of our reviewers stated, lectures already present a challenge for many students, including people with visual, hearing and note taking impairments, as well as concentration limitations, and social anxieties. The move to online lectures presents an opportunity to increase accessibility in many cases and for some students not having to physically navigate university spaces may be a significant benefit. A further consideration in due course is that students who lip‐read are likely to face difficulties with mask‐wearing. However, the modification of teaching materials is a major task that will be unlikely to be completed by September 2020 but is more likely to be an ongoing endeavor over several years.

## 3 LABORATORY CLASSES: IMMEDIATE CHANGES

Laboratory classes are a fundamental component of most STEM disciplines, including ecology (e.g., Beck & Blumer, 2012). In many cases, laboratory classes are closely linked to fieldwork sessions and they are often directly linked to coursework. The immediate impact of COVID‐19 on many laboratory classes may not have been as severe as it could have been during the 2019/20 academic year because many laboratory classes had completed before restrictions were imposed in the UK and Ireland. This of course would not have been the case for institutions who moved online earlier in the semester, or with more of the semester remaining, as highlighted by one of our reviewers. However, for many institutions approximately 75% of the teaching academic year (e.g., excluding exams and study weeks) was completed by the time universities moved online, but this coming academic year, universities are likely to struggle to provide laboratory access on the usual scale.

Our online poll supports this with 53% stating that a response to laboratory classes was not applicable to them, suggesting either that laboratory classes were not part of the modules they were running at the time or that laboratory classes were complete. Of those who were running laboratory classes at the time, 17% (5 individuals) canceled them outright, 13% (4 people) shifted to tutorial‐style sessions, 13% recorded themselves undertaking the laboratory for students, 13% provided video links of the techniques to students, 7% (two people) provided “do it at home” options for students, and 7% live‐streamed themselves undertaking the laboratory so students could join the session online. Moving laboratory classes online is more complex than pivoting lectures. One respondent noted that it “took two to three full day's [sic] work to move a single 3‐hr laboratory online.” Reduced experience of the laboratory environment could have significant impacts on student learning and enthusiasm about ecology and other STEM disciplines as it is a components that students enjoy and engage with (Beck & Blumer, 2012). Short‐term cancelation or replacement with videos/remote teaching is unlikely to have a major impact, but longer‐term a more refined solution will be required.

### 3.1 Changes for next year and beyond

Where face‐to‐face classes are run it will not be at the same capacity as usual due to social distancing measures imposed by governments. Therefore, many laboratory classes are likely to be online next year with those going ahead face to face being reduced. Therefore, many laboratory classes are likely to be online next year with those going ahead face to face being greatly reduced due to social distancing measures imposed by governments.

Laboratory classes are clearly more challenging to move online than lectures, and although some interesting resources (e.g., www.lecturemotely.com/copy‐of‐lab‐courses) exist to help guide nonlaboratory or remote laboratory class development, this represents a major challenge for teaching staff in the next academic year. Our online poll respondents did not converge around similar plans, highlighting the complexity of trying to move laboratories online. Some intend to attempt “do‐at‐home” laboratory sessions for students, use of live broadcasting or prerecorded videos of staff undertaking the laboratories. No one solution was mentioned by more than three respondents. Simple do‐at‐home laboratories may be helpful for students in earlier years of degree programmes where many laboratory procedures may be possible at home (e.g., extraction of DNA from fruit, some basic plant, and animal identification) but will likely become more difficult with more advanced classes, due to health and safety and the specialized equipment needed. For ecology, as one reviewer suggested, student led field investigations near the students’ home are possible, which can be complex and advanced, without specialist equipment. The effort from the lecturer comes in helping students develop an appropriate project and supporting data analysis. This is an advantage for ecology for keeping students engaged with field learning if usual field classes are not available. However, a note of caution does need to be applied here with respect to safety and risk assessment of activities. Laboratory classes did emerge as a source of concern for the coming year with one respondent noting “Lack of labs is particularly worrying.” Virtual laboratories, video recordings, and lecturers recording themselves doing the laboratory are also options that some are considering and offer useful stop‐gap measures. The benefits of these possibilities include the students still seeing the laboratory session and learning the procedures but the costs are the lack of physical experience in the laboratory environment and increased work load for staff. Although there are ways to address the reduction in laboratory experience, see Youssef et al. (2020) and lecturemotely www.lecturemotely.com/copy‐of‐lab‐courses for examples, poll respondents indicated that this is the most challenging component to address robustly via online or virtual methodologies.

## 4 FIELDWORK: IMMEDIATE CHANGES

Higher Education Institutes reacted differently to fieldwork provision when COVID‐19 restrictions were first implemented. A range of responses was evident across various institutions, including socially distanced field sessions to virtual field trips, but the main response seemed to be cancelation (14 respondents) or shifting to virtual field classes of one form or another (6 respondents) (Figure 1). In many cases, cancelation of planned field trips (9 respondents) was the only realistic option due to the rapid nature of the response required. In the NUIG Ecology & Conservation module a planned field trip to one of the conservation centers in the Burren, West of Ireland) was canceled because the center itself decided to close due to the pandemic in advance of the government‐mandated lockdown. An alternative field trip was briefly considered but not pursued because of time constraints, and ultimately, this would not have been permitted as the country was in lockdown on the planned date of the field trip. In the University of Leeds, planned second‐year residential field trips were also canceled for the 2019/20 academic year, as these would have violated government guidelines.

Not all UoL fieldwork was canceled. In a large first year module “Planet Under Threat,” an urban ecology practical was redesigned so students could conduct the tasks in their own gardens or homes, complying with lockdown regulations and students shared their work via Twitter (#GEOG1000Eco). Lecturers tweeted and replied to student tweets over the course of one working day. Only a low percentage of students engaged, but this field trip had become optional, and other students joined in later, or emailed through their work. This type of fieldwork is an extension of the move withing Ecology teaching toward more Living Lab work as reviewed by Cooke, Araya, et al. (2021) and Cooke, Wheeler, et al. (2020), and sharing data from different locations could extend the use of this set up.

Virtual field classes enable students to experience otherwise inaccessible locations (Cliffe, 2017) and offered a good replacement option for in person field classes in the pandemic, particularly if they are already available, and also have much to offer for longer‐term use. In many cases, subjects such as physical geography, geomorphology, or geology may be able to easily use freely available resources such as Google Earth or Google Engine to study various environments and geological/geomorphological features (e.g., https://serc.carleton.edu/NAGTWorkshops/structure/approach.html; Lamb & Johnson, 2010; Lisle, 2006). However, virtual fieldwork will not always be suitable or possible and Cooke, Araya, et al. (2021) and Cooke, Wheeler, et al. (2020) state virtual fieldwork should not replace traditional teaching but complement it. Some respondents noted that virtual replacements for fieldwork did not work well in all cases “Some students were left behind” and “the field course was never the same without the field…” However, as one reviewer pointed out, comparing virtual replacements for fieldwork that were put together quickly and in an emergency situation really cannot be compared to those that have been planned and developed over time. Virtual field trips have been shown to work very well when they are developed with a clear pedagogy and with clear aims and learning outcomes in mind (e.g., Markowitz et al., 2018.

For ecology, using currently available virtual resources is more challenging. There are few (if any) freely available resources to quadrat plants or sample insects for example. While some are in development, this existing gap meant that for many canceling field trips was the only realistic option. Although individual institutions may have resources suitable for virtual teaching of ecology, these are not currently clearly available in the public domain and likely remain closed IP of individual institutions and not available in many others. Several respondents raised concerns around this point “Students also struggled with understanding the finer material presented in field trip videos (e.g., identifying plant characters).” The replacement of field trips with videos and discussions was also not always particularly successful or popular with students: “students had to watch videos of cockroaches on YouTube, extract data and then write it up. Understandably they hated it and the write‐ups were really poor as they did not engage.” It should be highlighted, as one reviewer commented, that the skills required to put successful online and virtual teaching material together and specialist ones that many experienced lecturers are likely to lack. Many university lecturers are now learning these skills but it is not realistic to expect lecturers new to online pedagogy and teaching design to have the same skill set as those with years of experience in online teaching.

### 4.1 Changes for next year and beyond

Fieldwork is often a core component of ecology, botany, zoology, and physical geography degrees and usually a popular method of learning among students (Boyle et al., 2007; Dresne et al., 2013). Inability to provide in situ field experience is a major challenge to teaching staff as this is not easily replicated online. There are a range of increasingly good virtual fieldwork tools that can be used to meet field‐related learning outcomes freely available online (e.g., virtual glaciers and glaciated landscapes (https://vrglaciers.wp.worc.ac.uk/wordpress/ ) and virtual landscapes ( https://www.see.leeds.ac.uk/virtual‐landscapes/) and work to collate and fill gaps is ongoing both on an individual basis and in some newly developed international collaborative efforts (e.g., Virtual Palaeoscience (https://virtualpalaeoscience.wordpress.com/; palynology short talks (https://palynology.org/palynology‐short‐talks/) and #FieldWorkFix on Twitter). However, other areas such as vegetation identification and animal behavior are far more challenging and lacking in publically available online resources. For plants, the resolution of photographs or videos is not always clear enough to make identification easy; plants often need to be physically turned or touched to aid identification; and in other cases, a hand lens or even microscope and a physical sample may be required for a clear identification. Two respondents and the authors intend to produce relevant and tailored videos to aid in fieldwork teaching in the next academic year, but did not specify if these resources will be made publically available, it is the intention of the author to make vegetation quadrat videos publically available when they are produced.

Similar to laboratory work, there was no clear consensus among our respondents on how to approach the challenges associated with fieldwork in the COVID‐19 era. Some fieldwork is possible in a “do it yourself” or “do it at home” manner, and one respondent highlighted this a planned response. For example, asking students to count the number of birds they see from a window, garden or balcony for an hour (e.g., similar to the Great British Birdwatch) or collect, describe, and identify three or four plants (subject to local conservation laws and landowner permission) should be relatively straightforward tasks regardless of where students may be living. Pooling class data could provide very interesting data sets for comparison, for example, between national regions, between urban and rural living students (see Cooke, Araya, et al., 2021; Cooke, Wheeler, et al., 2020 for further ideas). Techniques such as these are already used in many higher education institutions, but are not widely represented in the literature.

One possibility that was only alluded to (and not specifically stated) by two respondents but is a planned response by the authors is the moving of fieldwork onto the university campus. Many universities are moving toward increased utilization of their campuses, for example, Leeds Living Lab (https://sustainability.leeds.ac.uk/the‐living‐lab/) and NUIG Community and University Sustainability Project (http://www.nuigalway.ie/sustainability/learn/). This may make quite a lot of fieldwork still possible in the next academic year. This approach has the benefit of ensuring some field experience for students in a generally safe environment where they can work socially distanced in small groups or pairs, and it will also enhance the use of university campuses (Bacon & Peacock, 2016; Peacock & Bacon, 2018; Peacock et al., 2018). This type of approach, while best suited to universities with large grounds can also be applied in universities with less available space with a focus on urban ecology (Bacon & Peacock, 2016). University campuses are likely to be able to facilitate many common ecological techniques including vegetation surveying; tree height/biomass assessment; interpreting animal traces; habitat assessment among others. Such field activities could be linked to a tutorial session to discuss methods, provide student feedback, and discuss outcomes. This will have the benefit of students not missing the opportunity to develop field skills, and it may have the further benefit of reduced novelty effect. The approach, however, will mean access to a limited range of ecosystems and new sites. However, many ecologists already run successful campus‐based field activities (Bacon & Peacock, 2016; Peacock et al., 2018; Savanick Hansen, 2017; Savanick et al., 2008) and extending these to a wider range of ecological activities will enhance use of the campus as a resource. Additionally, as stated by one of our reviewers, approach has been taken by many institutions, notably The Open University in the UK. Many universities worldwide have been offering online options for years, with simultaneous delivery to on‐campus and distance students completely normal for some, including for biology and ecology modules.

## 5 FURTHER PERSPECTIVES

The sudden move to online teaching and learning triggered by the COVID‐19 pandemic is unprecedented in higher education. Even plans for next academic year can be considered an emergency response as it is not possible to fully redesign courses in a few months. Challenges associated with this move featured considerably in our online poll “Online delivery is completely different to in‐class delivery and the summer is too short to completely reorganise all my teaching methods, delivery, practicals and field trips.” For ecology and aligned disciplines the major concerns relate to the loss of learning with reduced (or removed) fieldwork opportunities. Respondent concerns relating to the loss or reduction of fieldwork included, “I think that the fieldwork aspect of Ecology is vital to what we do and many students haven't had an opportunity to get taught some of the necessary [skills]” and “Field work will likely be a shadow of original field work teaching due to limitations of bringing large groups to relatively remote places.” The challenge of maintaining fieldwork and learning associated with fieldwork will remain a challenge for many in the coming academic year. This major concern perhaps explains why nearly 50% of our online poll respondents felt that these disciplines are more hampered that even other STEM disciplines at the present time. The range of concerns expressed by our online poll respondents is summarized in Figure 2.

A more general but still significant concern raised was that some universities are “overprescribing” how staff should be teaching while also significantly increasing staff workloads “Literally I haven't enough hours in the week to meet the demands of what my institution has decided, I also think that our uni has been too prescriptive and reduced the chance for decent reflection of how we can make this work online.” Another respondent highlighted that some universities are expecting too much and making too many promises to students. “I think people need to accept that we cannot provide the same level of teaching as we were doing pre COVID for various reasons. Burn‐out from staff and disillusionment from students are very likely if too many promises are given…”. This is something that universities need to consider in terms of its teaching staff—the role of a university lecturer is often highly demanding and stressful already (Morrish, 2019) and changes to the job that result in staff feeling less fulfilled and experiencing increased loneliness will exacerbate mental health issues within the profession. However, several respondents also found the positive in the situation and noted that they were pleased to be able to review courses and assessments and learn new skills “I am happy to have been forced to learn so much about the tools available” and “This provided us an opportunity to think about what forms of assessment best develop skills that students will need in the workplace.**”**

In conclusion, the challenges of both laboratory‐ and field‐based teaching and learning in ecology and aligned disciplines present a series of challenges to teaching staff. Learning new methods, developing new resources and implementing them effectively is a challenge, particularly in institutions where online learning is a generally new feature for most lecturers. Training and support for lecturers is needed and the timescale of these changes also present a challenge to university staff. However, the availability of technology offers at least a partial answer and innovative pedagogic solutions are actively being pursued across the discipline.

## CONFLICT OF INTEREST

The authors declare there are no conflicting interests.

## AUTHOR CONTRIBUTION

**Karen L. Bacon:** Conceptualization (equal); Investigation (equal); Methodology (equal); Project administration (equal); Resources (equal); Validation (equal); Visualization (equal); Writing‐original draft (equal); Writing‐review & editing (equal). **Julie Peacock:** Conceptualization (equal); Formal analysis (equal); Investigation (equal); Methodology (equal); Resources (equal); Software (equal); Validation (equal); Visualization (equal); Writing‐original draft (equal); Writing‐review & editing (equal).

## Supporting information

Supplementary Material

## Data Availability

Survey data that support the findings of this study are available in the Supporting Information of this article. All individually identifying information has been removed, and long‐form answers have not been shared because informed consent to do so was not obtained from respondents.
